# Frequency of MicroRNA Response Elements Identifies Pathologically Relevant Signaling Pathways in Triple-Negative Breast Cancer

**DOI:** 10.1016/j.isci.2020.101249

**Published:** 2020-06-06

**Authors:** Asha A. Nair, Xiaojia Tang, Kevin J. Thompson, Peter T. Vedell, Krishna R. Kalari, Subbaya Subramanian

**Affiliations:** 1 Department of Health Sciences Research, Mayo Clinic, 200 First Street SW, Rochester, MN 55905, USA; 2 Department of Surgery, University of Minnesota, 420 Delaware St SE, Minneapolis, MN 55455, USA; 3 Masonic Cancer Center, University of Minnesota, Minneapolis, MN 55455, USA; 4 Center for Immunology, University of Minnesota, Minneapolis, MN 55455, USA

**Keywords:** Biological Sciences, Bioinformatics, Cancer Systems Biology, Cancer

## Abstract

Complex interactions between mRNAs and microRNAs influence cellular functions. The mRNA-microRNA interactions also determine the post-transcriptional availability of mRNAs and unbound microRNAs. MicroRNAs binds to one or more microRNA response elements (MREs) located on the 3′UTR of mRNAs. In this study, we leveraged MREs and their frequencies in cancer and matched normal tissues to obtain insights into disease-specific interactions between mRNAs and microRNAs. We developed a bioinformatics method “ReMIx” that utilizes RNA sequencing (RNA-Seq) data to quantify MRE frequencies across the transcriptome. We applied ReMIx to triple-negative (TN) breast cancer tumor-normal adjacent pairs and identified MREs specific to TN tumors. ReMIx identified candidate mRNAs and microRNAs in the MAPK signaling cascade. Further analysis of MAPK gene regulatory networks revealed microRNA partners that influence and modulate MAPK signaling. In conclusion, we demonstrate a novel method of using MREs in the identification of functionally relevant mRNA-microRNA interactions in TN breast cancer.

## Highlights

• Bioinformatics method ReMIx identify differential microRNA response rlements (MRE)
• Tumor-specific MREs frequency observed in triple-negative breast cancer (TNBC)
• MRE analysis identify MAPK signaling genes as therapeutic target for TNBC
• MREs frequency can be used to identify pathologically relevant pathways

## Graphical Abstract

**Figure:**
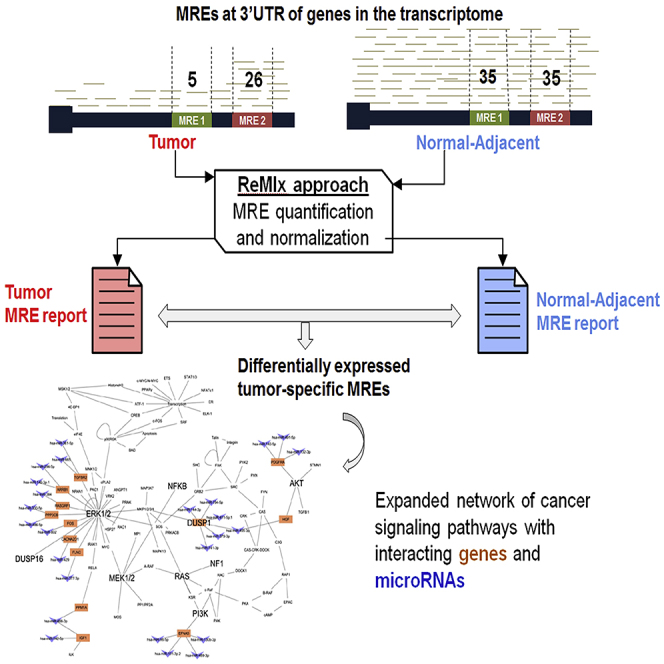

## Introduction

Regulatory interactions between coding and non-coding RNAs in cells determine the post-transcriptional availability of protein-coding mRNA transcripts (Chiang et al., 2010, Eichhorn et al., 2014, Garcia et al., 2011, Guo et al., 2010, Guo et al., 2014, Lee and Jiang, 2017, Rissland et al., 2017, Shin et al., 2010, Volinia and Croce, 2013, Wu and Bartel, 2017). MicroRNAs use seed sequences (6–8 bases long) to bind to microRNA response elements (MREs) predominantly located on the 3′UTRs of mRNAs. mRNAs can have one or more distinct MRE sites, thus being targets to multiple microRNAs. Similarly, microRNAs also bind to MRE sites of several different target genes (Krek et al., 2005, Lim et al., 2005). Thus, alterations in target gene expression via microRNA binding can affect several cellular processes such as cell proliferation and apoptosis during cancer development, progression, and metastasis. Thus, elucidating critical players among the mRNA-microRNA interacting networks can yield novel therapeutic targets and biomarkers in cancers, especially for cancer subtypes that are least responsive to current modalities of treatment.

Expression profiles of microRNAs and mRNAs (Illumina TruSeq libraries enriched for poly(A) RNAs) across many cancer types in The Cancer Genome Atlas (TCGA) were used to infer active and functional microRNA-target interactions in different cancer types (Jacobsen et al., 2013). Alternative polyadenylation of 3′UTRs in bladder cancer can lead to shortened 3′UTR affecting mRNA stability and attenuated protein translation (Han et al., 2018). Studies have also shown that the presence of single nucleotide polymorphisms (SNPs) in the 3′UTR of transcripts can affect microRNA binding and are associated with multiple cancer subtypes (Pelletier and Weidhaas, 2010). Here we extrapolate TCGA RNA sequencing (RNA-Seq) data to analyze MRE sites to obtain insights into unique interactions between mRNAs and microRNAs at the 3′UTRs of the tumor and normal-adjacent datasets.

We developed a new bioinformatics approach called ReMIx (pronounced “remix”)—m**R**NA-**M**icroRNA **I**ntegration—which leverages RNA-Seq data to quantify MRE sites at the 3′UTR sequence across the transcriptome. ReMIx profiles MRE sites in tumor and matched normal samples separately, which enables the identification of differential frequency of MREs that are statistically significant in tumor samples. Because MRE is the interacting link between mRNAs and microRNAs, ReMIx brings together mRNAs with tumor-specific MREs and microRNAs that have the potential to bind to these MRE sites. ReMIx also reports potential mRNA-microRNA candidates that have unique tumor-specific interactions and potential disease-driving functions. This method can be applied to study any cancer type or complex diseases along with their normal tissue sets. To demonstrate the utility of ReMIx, we applied it to the largest RNA-Seq dataset of breast cancer cases and normal-adjacent tissues from TCGA (Cancer Genome Atlas Network, 2012, Ciriello et al., 2015). Using this method, we specifically identified MREs in estrogen receptor positive (ER+), ErbB2 overexpressed–HER2 positive (HER2+), triple-negative tumors, and normal-adjacent tissues. Triple-negative breast cancers (TNBC) are highly heterogeneous and one of the most severe forms of breast cancer subtypes with no targeted treatments currently available. In this study, we applied ReMIx and identified mRNA-microRNA candidates unique to the TNBC and not present in ER + or HER2+ subtypes. Analysis of TNBC data identified MAPK signaling pathway targets as a potential disease driver and target.

## Results

### ReMIx: An Automated Bioinformatics Approach for MRE Quantification

**Figure 1:**
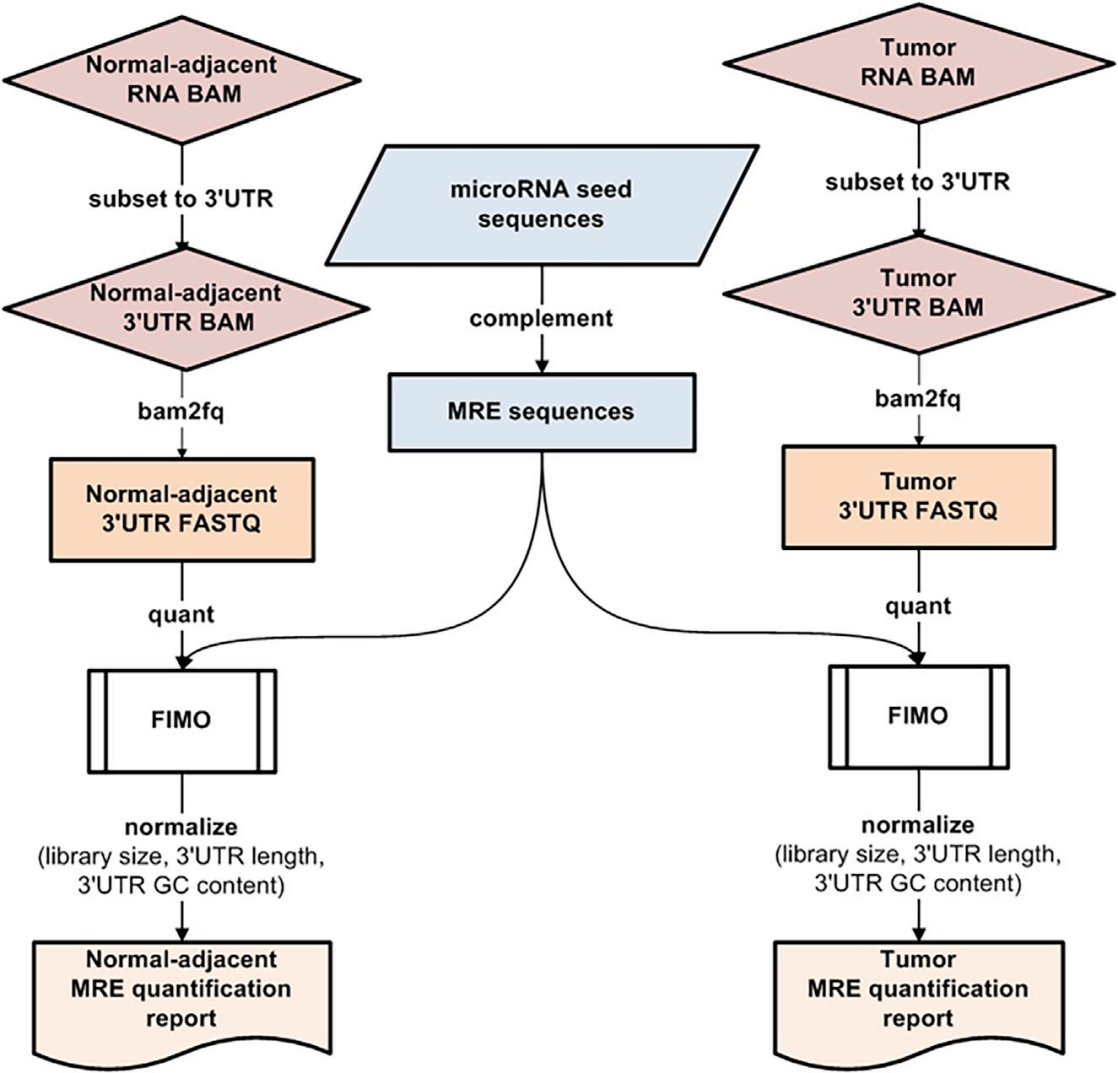
Flowchart of MRE Frequency Quantification from RNA-Seq BAM The RNA-Seq BAM is subset to 3′UTRs of all genes, converted to FASTQ and processed through FIMO to obtain raw MRE counts per microRNA for every target gene. The raw MRE counts were normalized to account for library size, 3′UTR length, and 3′UTR GC content, and individual tumor and normal-adjacent quantification reports are generated. See also Data S1, S2, and S3.

We developed an innovative bioinformatics approach called ReMIx to quantify the expression of MRE sites at the 3′UTRs of mRNAs using RNA-Seq data. ReMIx uses reads aligned to 3′UTRs of genes in a given transcriptome and scans them for evidence of MRE sequences (see Transparent Methods). All known MREs for genes in the reference genome, as reported by TargetScan—human version 7.0 (Agarwal et al., 2015), are quantified for their level of expression at the 3′UTR of all genes. After quantification, ReMIx normalizes the raw counts of MREs to account for sample library size, 3′UTR length, and 3′UTR GC content per gene. Finally, for every gene and for every conserved microRNA that targets the gene, the normalized MRE counts are reported in a tab-delimited format for each gene-microRNA pair in the transcriptome analyzed. The ReMIx workflow is fully automated and designed to run in a multithreaded cluster environment to analyze paired-end transcriptome samples. A flowchart of the ReMIx approach is shown in Figure 1 (see Transparent Methods).

### ReMIx Identified 221 Triple-Negative Breast-Cancer-Specific MRE Sites

The 3′UTR sequences of individual genes (n = 12,455, TargetScan v7.0 (Agarwal et al., 2015)) were obtained using the reference human genome hg19 build. Reads aligned to these 3′UTR sequences were obtained using the TCGA Breast Cancer transcriptome dataset for 13 pairs (Tumor and Normal-Adjacent) from the TNBC subtype, 56 pairs of ER+, and 20 pairs of HER2+ subtypes and were provided as input to the ReMIx workflow (see Transparent Methods). The pre-computed MRE sequences (n = 329, TargetScan 7.0) were also provided as input to ReMIx to count reads mapped to individual MREs located on each gene. The raw MRE counts were then normalized by factoring library size, 3′UTR lengths, and 3′UTR GC content of individual genes. MRE quantification process identified normalized counts of 111,521 MRE sites in tumor and normal adjacent sample sets for each subtype (Data S1, S2, and S3 for TN, ER+, and HER2+, respectively).

**Figure 2:**
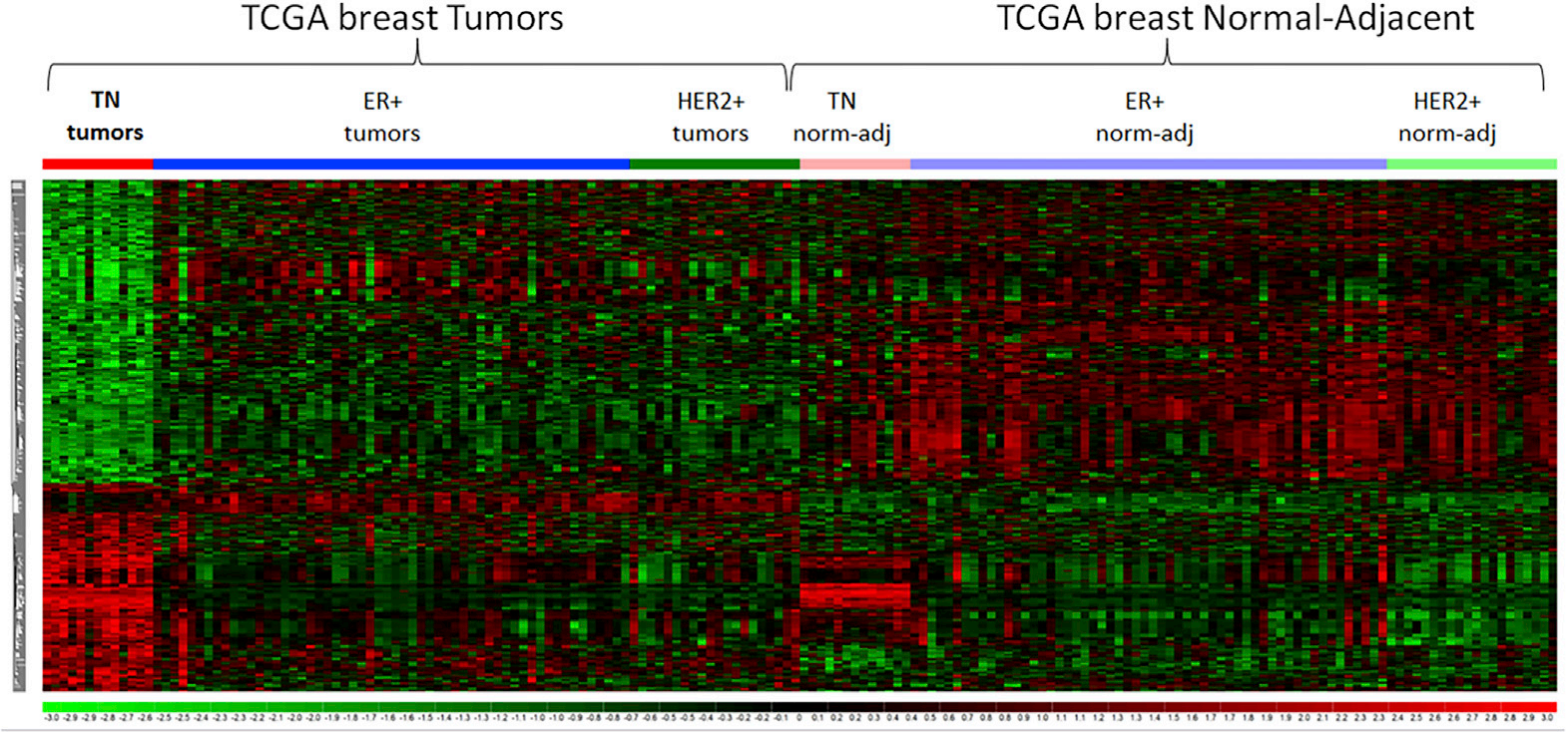
Heatmap of 221 TN Tumor-Specific MREs The normalized conditional quantile normalization (CQN) values of 221 MREs were obtained for TN, ER+, and HER2+ tumors and normal-adjacent (norm-adj) cases. As shown in the heatmap, these MREs have a distinct expression in TN tumors in comparison to the other subtypes as well as TN normal-adjacent cases. See also Figure S1; Data S1, S2, S3, S4, S5, S6, and S7.

Next, ReMIx results were used to identify MRE sites that had unique and significant levels of expression (high or low) in TNBC tumors in comparison to ER + tumors, HER2+ tumors as well as TN, ER+, and HER2+ normal-adjacent cases. The Dunnett-Tukey-Kramer (DTK) pairwise multiple comparison statistical test was applied to the tumor and normal-adjacent cases across all subtypes (six groups in total) to highlight MREs that were unique only to TNBC (p value < 0.05) when compared with other two subtypes and all normal-adjacent cases. This resulted in identifying 614 MREs unique to TNBC (Data S4). In addition, the edgeR bioinformatics package (Robinson et al., 2010) was applied to identify differentially expressed MREs by comparing 13 TN tumor and the respective normal-adjacent cases (FDR <5% and log2FC |2|) and reported 3,053 significant and differentially expressed MREs (Data S5). By adopting the approach of taking the intersection of MREs reported to be statistically significant and differentially expressed by the two complementary approaches, i.e., DTK (n = 614 MREs) and edgeR (n = 3,053 MREs), we identified a common set of 221 TN tumor-specific MRE sites (Figure S1). The 221 TNBC MREs are provided in Data S6. The distinct expression profile of these MRE sites in TNBC with respect to other subtypes and normal-adjacent cases are shown in the heatmap (Figure 2).

### TNBC-Relevant MREs Are Associated with 88 mRNAs and 125 microRNAs

The unique feature of MRE is that it is the interactive site between mRNA and microRNA. Hence, for MRE sites of interest, we can decode and obtain information about the mRNA and its interacting microRNA by identifying the relevant MREs and decoupling them into their respective mRNA and microRNA pairs. Thus, for the TNBC tumor-specific MRE sites, we deciphered such information for the 221 MREs and obtained a total of 88 mRNAs and 125 microRNAs. Tables listing 88 mRNAs and 125 microRNAs along with their expression levels in TNBC are provided in Data S7 and S8, respectively. With these ReMIx analyses, we deduce that over half of the mRNAs (48 out of 88) were used repeatedly and these mRNAs had multiple MREs that were used as interactive sites by different microRNAs.

**Figure 3:**
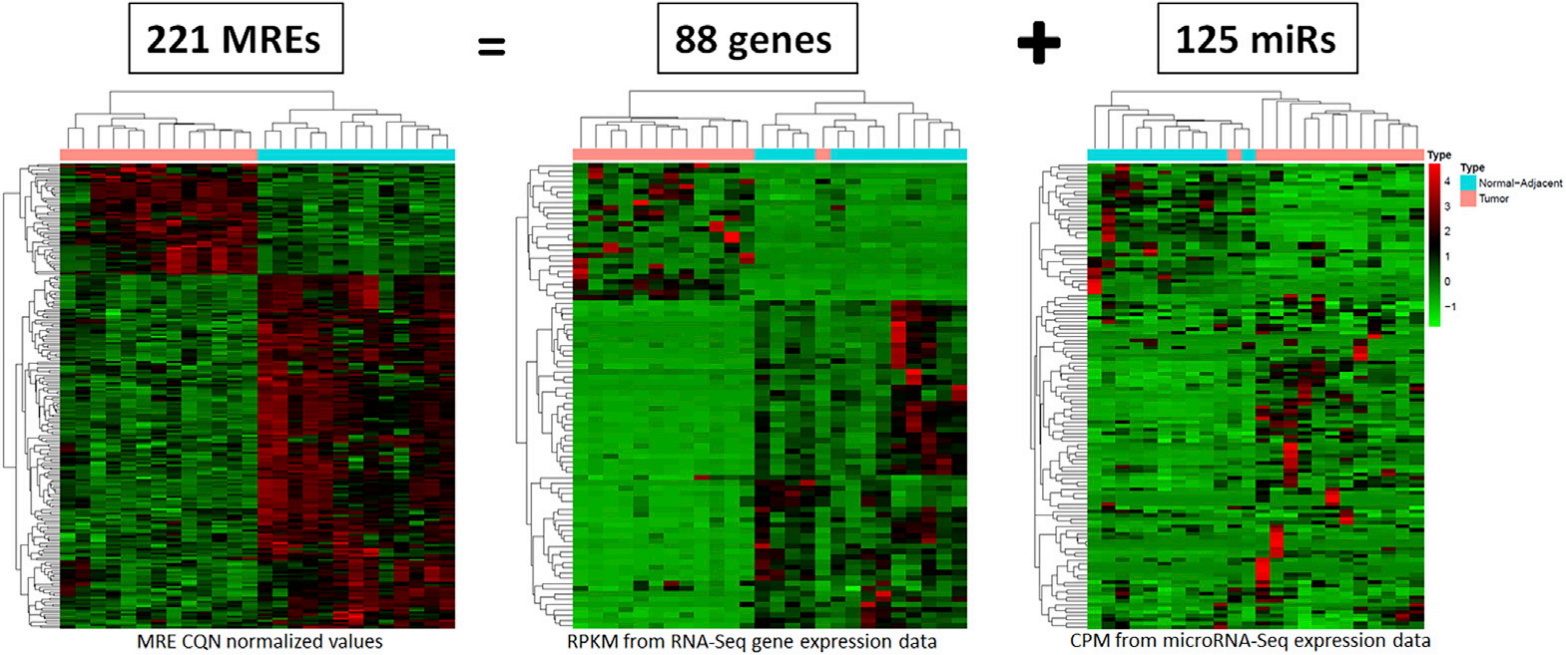
Unsupervised Clustering and Heatmap Representation of 221 TN Tumor-Specific MREs and Their Associated mRNAs and microRNAs The 221 MREs were associated with 88 mRNAs and 125 microRNAs. Conditional quantile normalization values were obtained for the MRE sites. Reads per kilobase per million mapped reads (RPKM) normalized values from RNA-Seq and counts per million (CPM) normalized values from microRNA-Seq were obtained for the 13 pairs of TN tumor and normal-adjacent cases. Unsupervised clustering of the cases indicates that tumor (pink) and normal-adjacent (blue) were clustered well within the corresponding groups. See also Figure S1; Data S7, S8, S9, S10, and S11.

Unsupervised hierarchical clustering of 221 MREs based on expression profiles of these MREs showed that the TN cases clustered within their tumor and normal-adjacent groups. The differential expression pattern for 221 MREs are shown in Figure 3. Notably, unsupervised clustering of the corresponding 88 mRNAs and 125 microRNAs also showed a separation of TNBC into tumor and normal-adjacent groups (Figure 3).

Next, we evaluated the mRNAs and microRNAs identified by ReMIx using a standalone approach to analyze their predominance in terms of differential expression within the respective RNA and microRNA expression datasets of 13 TNBC tumor and normal-adjacent pairs and identified canonical pathways that were associated with 88 mRNAs and 125 microRNAs.

The differential expression analysis using RNA-Seq data for 13 TNBC tumor and normal-adjacent pairs showed that a total of 2,250 genes were differentially expressed (edgeR package (Robinson et al., 2010); statistical significance threshold at FDR <5% and log2FC |2|). Notably, out of the 88 mRNAs identified by the ReMIx analysis, we found that 68 (77%) were also differentially expressed at the gene level between TNBC cases. This indicated a high likelihood of microRNA-mediated gene expression regulation resulting in their differential expression in TNBC tumors compared with their normal-adjacent counterparts. Notably, these 68 mRNAs found in the 13 paired TNBC cases were also consistently differential expressed in a larger cohort of 120 TCGA-TNBC and 13 normal-adjacent samples (Figure S2). This suggests that mRNA expression observed in a smaller sample size is potentially reflective of mRNA expression in a larger cohort. Out of 68 mRNAs, 41 had multiple MREs targeted by different microRNAs. A table listing these 68 mRNAs that are both differentially expressed and have interacting MRE sites are given in Data S9.

Next, using the microRNA expression data for 13 TNBC pairs, we found that out of a total of 2,245 microRNAs that were quantified for expression in the tumor and normal-adjacent cases, 778 microRNAs were differentially expressed in tumors (limma package (Ritchie et al., 2015); adjusted p value < 0.05). Examining the number of microRNAs identified by ReMIx that were also differentially expressed between TNBC tumor and normal-adjacent, we found that 64 out of 125 microRNAs (51%) were statistically different in expression (FDR <5%). A table and heatmap listing 64 microRNAs that are both differentially expressed and participate in MRE-mediated gene expression regulation can be found in the Data S10 and Figure S3.

Finally, using RNA-Seq and microRNA differential expression results, the magnitude and direction of change for 221 MREs and their associated genes and microRNAs in 13 TNBC tumor and normal-adjacent pairs were combined (Data S11). We observed that the majority of MREs follow the direction as their parent genes, with very few exceptions, likely due to the nature of TNBC sequencing libraries (Illumina TruSeq).

### MRE-Associated 125 microRNAs Are Implicated in TN Breast Carcinoma

Further analysis of 125 microRNAs using the TAM 2.0 tool for microRNA set enrichment analysis revealed that these microRNAs were associated with cancer pathways as shown in Table S1. Specifically, 14 out of 125 microRNAs are also reported in other TNBC studies and are upregulated with an FDR <2.87 × 10^−5^. Similarly, 55/125 microRNAs are reported in breast carcinoma studies (FDR <8.18 × 10^−18^) and 34/125 in breast neoplasms (FDR <6.12 × 10^−13^). Information of these microRNAs are provided in Table S1.

### Pathway Analysis of 88 Genes Identified MAPK Signaling Pathway

Eighty-eight genes obtained from ReMIx were analyzed to identify their associated signaling pathways. Using gene set enrichment analysis (GSEA) (Subramanian et al., 2005) on KEGG and REACTOME databases, the mitogen-activated protein kinase (MAPK) signaling cascade was identified among the top significant pathways. In addition, application of the signaling pathway impact analysis (SPIA) package also confirmed that the MAPK signaling pathway was activated in TN tumors. The GSEA and SPIA pathway results are provided in Data S12 and S13, respectively.

**Figure 4:**
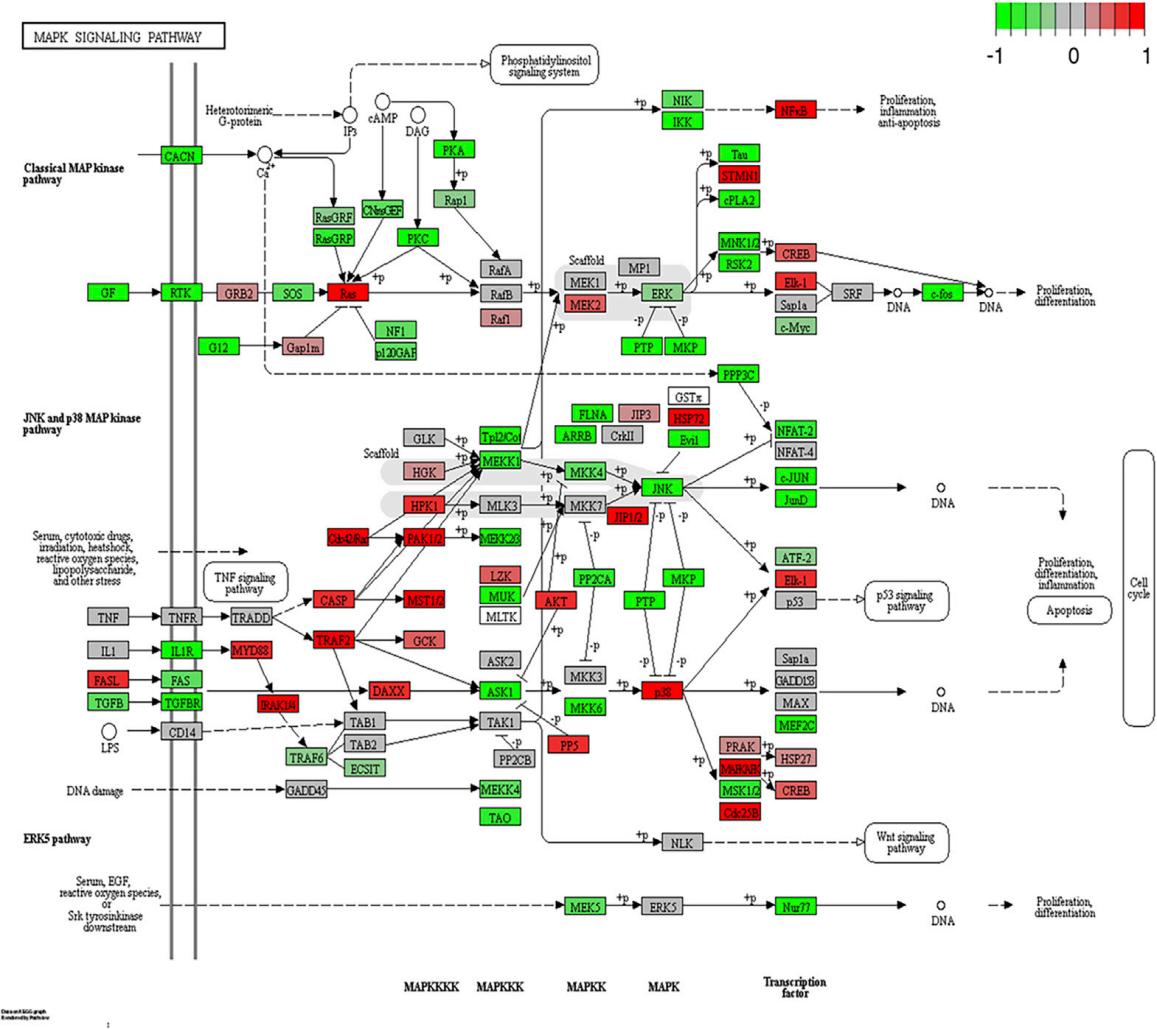
MAPK Signaling Pathway Genes in the pathway are colored based on their expression in TN tumors. Oncogenes NFKB and AKT are activated in this pathway. See also Data S12 and S13.

Further examination of genes in the MAPK pathway was conducted by juxtaposing the expression of these genes, obtained from RNA-Seq data of TNBC with the KEGG-based network of the MAPK pathway. Our analysis revealed that oncogenes *KRAS, NRAS, AKT*, and *NFKB* were notably activated and tumor suppressor *PTEN* was repressed. Figure 4 illustrates the KEGG pathview for the MAPK signaling cascade. MAPK signaling pathway is an extensive cascade with connections to several biological pathways downstream such as proliferation, cell cycle, glycolysis, apoptosis, and protein synthesis.

### Expanded MAPK Endogenous RNA Network including TNBC-Specific mRNA-microRNA Candidates

**Figure 5:**
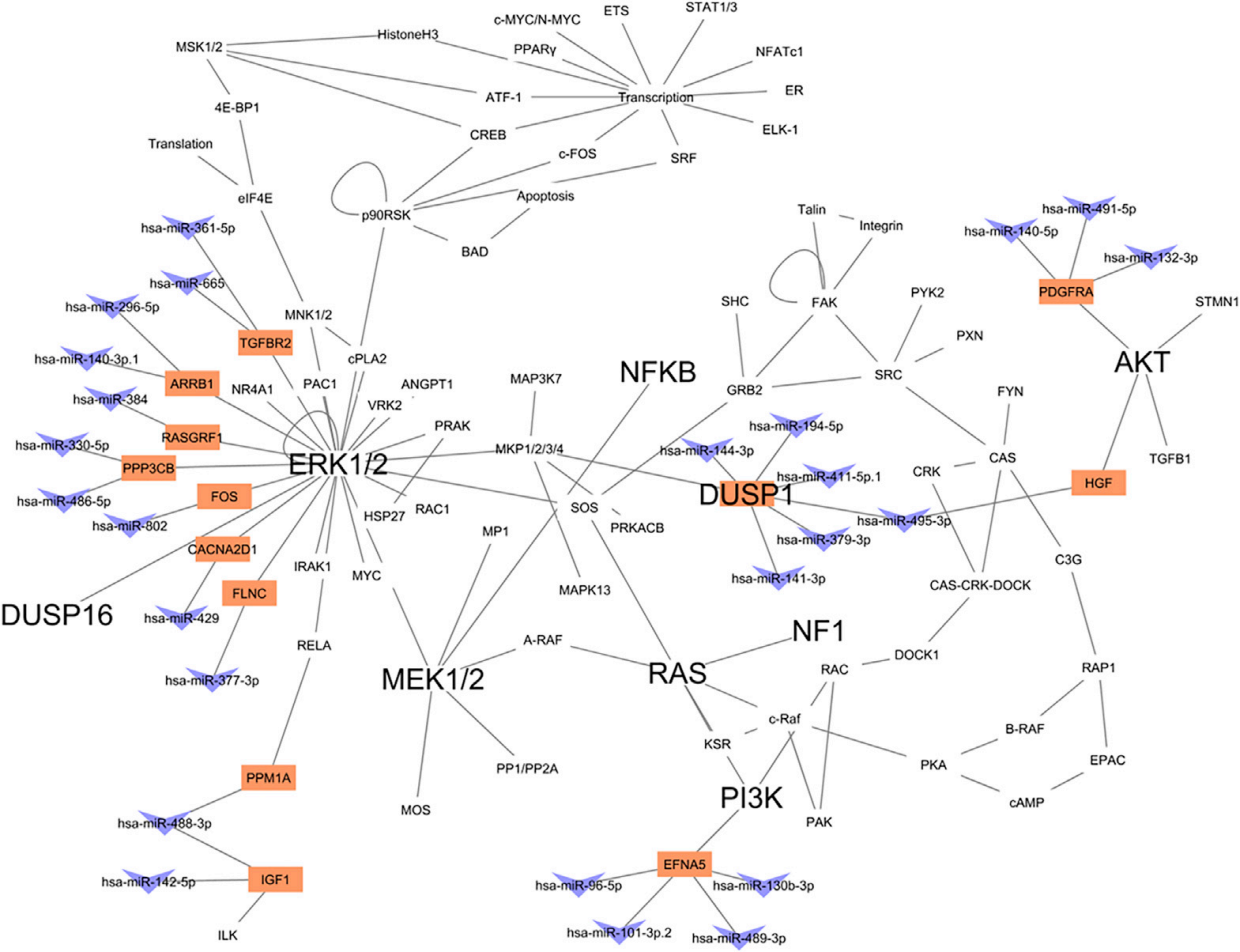
MAPK Endogenous RNA Network This figure shows the network of interacting protein-coding mRNAs and non-coding microRNAs in the MAPK singling pathway. The mRNAs and microRNAs reported by ReMIx are represented in colors orange and blue, respectively. Oncogenes AKT, RAS, NFKB, PI3K, ERK, and MEK are shown to interact either directly or indirectly with the mRNA-microRNA candidates. See also Table 1.

Based on MRE results from the ReMIx, we investigated relevant genes that were associated with MAPK signaling in TN tumors. We found 12 out of 294 genes (~4%) in MAPK pathway (https://www.genome.jp/dbget-bin/get_linkdb?-t+genes+path:hsa04010) that have MRE sites with the potential of differential binding of microRNAs. These 12 mRNAs with tumor-specific MRE sites and microRNAs with the potential to bind to these sites are provided in Table 1. Next, we expanded the MAPK gene network in TN tumors by including interacting microRNAs that are essential members of the pathway. Figure 5 shows the MAPK endogenous RNA network that represents the genes identified by ReMIx, interacting microRNAs, and other mRNAs that are likely to interact with each other and regulate expressions of key genes, such as *PI3K, AKT, RAS, NFKB,* and *PTEN*. Furthermore, *ERK1*/2, critical genes in the MAPK/ERK signaling cascade, were also directly associated with 7 of the 12 genes (Figure 5). Taken together, we present an expanded network of MAPK signaling cascade and provide a list of potential mRNA-microRNA candidates that interact with each other and could potentially be therapeutic targets for TN tumors.

**Table 1:** Gene-microRNA Pairs with Distinct TN-Specific MRE Sites that Are Part of the MAPK Pathway

mRNAs	microRNAs
**MAPK Signaling Pathway**	

*CACNA2D1*	hsa-miR-429
*PPP3CB*	hsa-miR-330-5p; hsa-miR-486-5p
*RASGRF1*	hsa-miR-384
*IGF1*	hsa-miR-142-5p; hsa-miR-488-3p
*HGF*	hsa-miR-495-3p
*EFNA5*	hsa-miR-101-3p.2; hsa-miR-130b-3p; hsa-miR-489-3p; hsa-miR-96-5p
*PDGFRA*	hsa-miR-132-3p; hsa-miR-140-5p; hsa-miR-491-5p
*FOS*	hsa-miR-802
*TGFBR2*	hsa-miR-361-5p; hsa-miR-665
*FLNC*	hsa-miR-377-3p
*ARRB1*	hsa-miR-140-3p.1; hsa-miR-296-5p
*PPM1A*	hsa-miR-488-3p

Table lists genes that are a subset of the 88 genes obtained from ReMIx and that are members of the MAPK signaling pathway. The microRNAs that bind to the MRE sites that were found to have distinct counts in TN tumors are also provided. Related to Figure 5.

## Discussion

The regulatory interactions between non-coding and protein-coding RNAs have been well recognized, where the mRNA-microRNA interactions are widely studied. Although there are several microRNA target prediction tools such as TargetScan (Agarwal et al., 2015), miRBase (Kozomara et al., 2019), DIANA (Vlachos et al., 2012), PicTar (Krek et al., 2005), miRwayDB (Das et al., 2018), miRanda (Betel et al., 2008), PITA (Kertesz et al., 2007), RNA22 (Loher and Rigoutsos, 2012), and miRTar (Hsu et al., 2011), not many computational tools have been developed that enable the integration of mRNA and microRNA expression datasets. MAGIA is a web-based tool for microRNA and gene integrated analysis that brings together target predictions and gene expression profiles using different functional measures for both matched and unmatched samples (Sales et al., 2010). The tool miRmapper uses mRNA-microRNA predictions and a list of differentially expressed mRNAs to identify top microRNAs and recognizes similarities between microRNAs based on commonly regulated mRNAs (da Silveira et al., 2018). HisCoM-mimi is a hierarchically structured component analysis method that models biological relationships as structured components to efficiently yield integrated mRNA-microRNA markers (Kim et al., 2018). These tools use prior knowledge of microRNA target predictions and are developed using unique methodologies to derive mRNA-microRNA interactions. Furthermore, tools such as miRmapper have the ability to highlight key microRNAs based on the number of connections it possesses in a given network. However, the underlying methodologies of all these tools are to use the expression of either mRNAs alone or both mRNAs and microRNAs to model their correlation and derive mRNA-microRNA relationships.

With the advent of RNA-Seq technology, profiling of the transcriptome is now possible at the base-prevision level. It is a known fact that microRNAs predominantly bind to the 3′UTRs of mRNAs to induce their regulatory effects and thereby impact mRNA expression and protein translation. Studies have shown that shortening of 3′UTR is a frequent phenomenon in cancer to evade oncogenes from microRNA suppression (Xue et al., 2018), repress tumor suppressor genes (Park et al., 2018), and enhance metastatic burden (Andres et al., 2019). Therefore, it is important not only to know which mRNAs are differentially expressed between a tumor and normal pair but also to determine which integration sites or microRNA response elements (MREs) are available along the 3′UTRs of the tumor mRNAs. Identification of MREs that are either present/absent/highly expressed/low expressed in the tumor can provide mechanistic insights of tumor progression. Although mRNA-microRNA integration tools exist, and may be applied to the tumor and normal datasets, no tools, to our knowledge, have the ability to precisely report mRNA-microRNA interactions that are solely based on the availability of MREs at the 3′UTRs. MREs are short 6–8 base segments and without appropriate bioinformatics methods, screening RNA-Seq data for MRE sites can yield highly non-specific and erroneous results. This could be a possible reason why this simple, but highly relevant, concept has not been explored to date.

**Figure 6:**
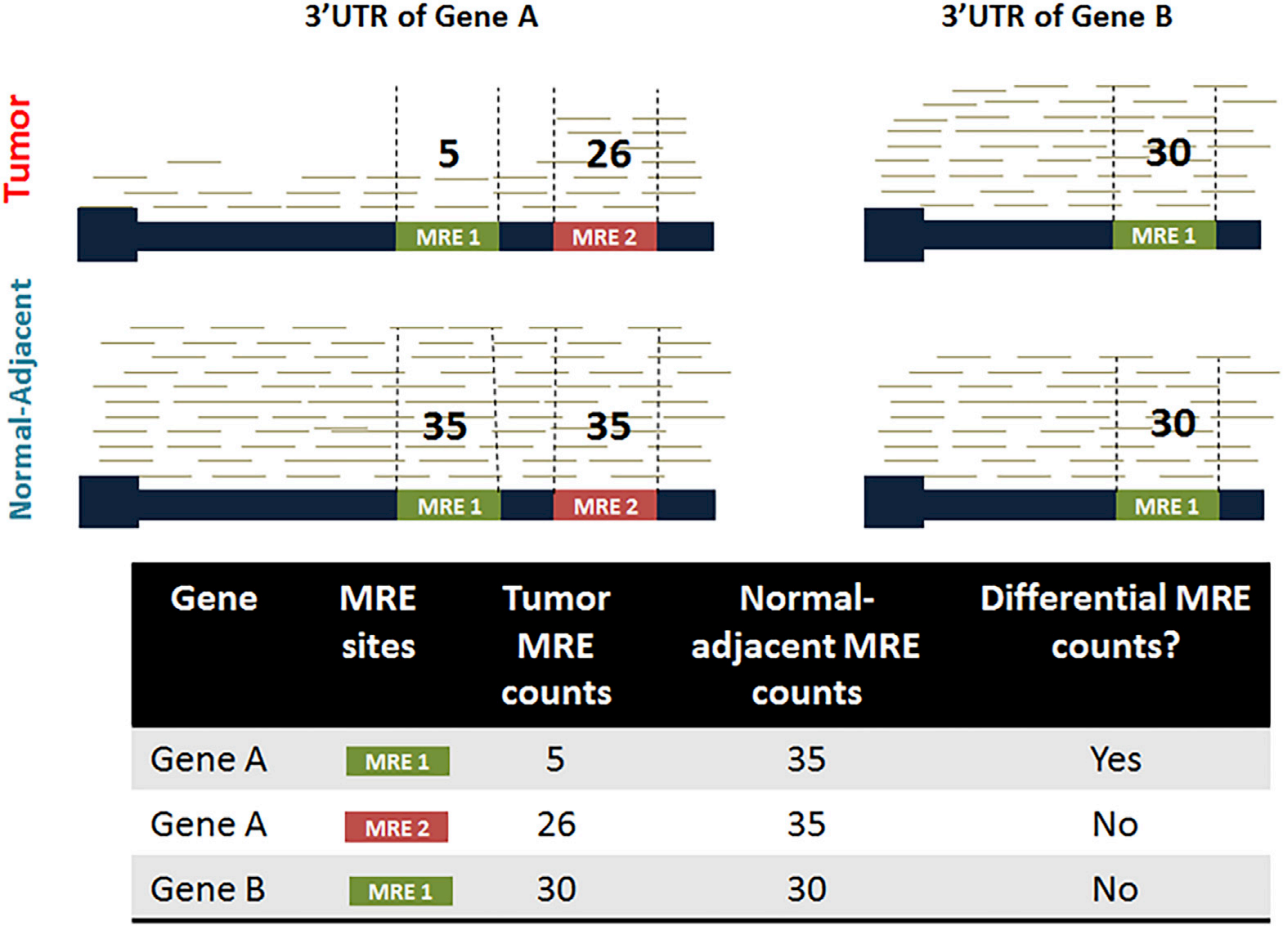
Hypothetical Representation of MRE Frequency Counting Using RNA-Seq Data The example shows a tumor and normal-adjacent sample with reads mapped to the 3′UTRs of two genes, Gene A and Gene B that consist of 2 and 1 MRE sites, respectively, with a common site (MRE 1). Reads that align with individual MRE sites (vertical dotted lines) are quantified. For every MRE site that belongs to a gene, the counts are then statistically evaluated between tumor and normal-adjacent for evidence of differential frequency (as shown in the inset table).

In this study, we developed an innovative bioinformatics method “ReMIx” that uses RNA-Seq data to identify and quantify microRNA-binding sites (known as microRNA response elements [MREs]) at 3′UTRs. A hypothetical example of this approach is illustrated in Figure 6. We applied ReMIx to TCGA-paired tumors and normal-adjacent breast cancer cases for TN, ER+, and HER2+ subtypes. Using two complementary statistical approaches, we identified 221 MRE sites that have a distinct expression in TN tumor-normal adjacent pairs. Upon decoupling, we found that the 221 MRE sites corresponded to 88 mRNAs and 125 microRNAs. By reviewing fold-changes of these MREs, mRNAs, and microRNAs, we observed that most of the MREs followed the same direction as their parent gene transcript. We postulate that this was likely driven by the sequencing library preparation kit (Illumina TruSeq). However, we also found MREs with the opposite trend, suggesting an alternative 3′UTR mechanism. Furthermore, we found mRNAs and MREs with positive expression in TNBC tumors but repressed microRNAs, likely denoting the effect of competing endogenous RNAs (ceRNAs) on the microRNAs. Canonical pathway analysis of both mRNAs and microRNAs revealed cancer-related pathways specific to breast cancer. Significantly, miR-27a and miR-143 were associated with breast cancer and TNBC, respectively (Jiang et al., 2018) (Deng et al., 2018). Upregulation of miR-27a induced epithelial-to-mesenchymal transition and increased cell migration in breast cancer (Jiang et al., 2018). Also, miR-143-3p was implicated in drug resistance; overexpression of miR-143-3p inhibits cytokine-induced apoptosis inhibitor 1 (CIAPIN1), enhancing the sensitivity of drug-resistant TNBC cells (Deng et al., 2018). Specifically, mRNAs revealed by ReMIx signified the MAPK signaling cascade in TNBC. The mRNAs determined by ReMIx represented about 4% of gene members in the MAPK signaling pathway. Based on TNBC-specific results reported by ReMIx, we expanded the MAPK endogenous RNA network by including mRNAs with TNBC-specific MRE sites. Further we also included the corresponding microRNAs that have the potential to bind to these MRE sites and other protein-coding RNAs in the network that have the ability to interact with each other and regulate expression of primary oncogenes and tumor suppressors in the MAPK signaling pathway. Based on the results, we provide a list of potential mRNA-microRNA candidates that interact with each other at the network level of the MAPK signaling cascade and could be possible therapeutic targets for TN tumors.

Because the TCGA breast cancer cases, including TNBC, have whole-exome sequencing data available, we sought to check for any copy number variant (CNV) events at the 3′UTRs. We also examined whether differentially expressed mRNAs, specifically those found by ReMIx, were related to CNV events. The metadata for exome capture kit for the 26 TNBC tumor and normal-adjacent cases was accessed using the NCI-GDC API (NCI Genomic Data Commons Application Programming Interface). For the majority of cases (18 out of 26), the “hg18nimblegenexomeversion2” capture kit was used. For the remaining 8 out of 26, “NimblegenEZExomev3.0” and “SureSelectHumanAllExon38Mbv2” capture kits were utilized for four cases each. Upon checking the overlap of these exome capture kits to 3′UTRs, we found that these kits do not cover the 3′UTR. We only observed a 0.4% overlap of 3′UTRs with “hg18nimblegenexomeversion2,” 13.8% with “NimblegenEZExomev3.0,” and 1.8% with “SureSelectHumanAllExon38Mbv2.” As a result, we could not verify the role of CNV on the TNBC cases for this study.

Recently, there has been an increasing focus to explore and identify therapeutic strategies to better treat and improve survival of TNBC patients. Activation of the MAPK pathway has been implicated in the proliferation and survival of cancer cells (Saini et al., 2013). Previous studies have shown this pathway to be highly prevalent in TN breast cancer as opposed to other breast cancer subtypes (Balko et al., 2012, Hashimoto et al., 2014, Hoeflich et al., 2009), thus supporting our findings. Studies have also shown that activation of MAPK pathway significantly correlates with disease progression in TN tumors (Eralp et al., 2008, Gholami et al., 2014, Giltnane and Balko, 2014, Hashimoto et al., 2014, Loi et al., 2016, Qi et al., 2015). MAPK pathway is a sequentially activated cascade consisting of key genes such as Ras, Raf, MEK, and ERK. Activation of Ras leads to the phosphorylation of Raf, thereby promoting the activation of MEK and ERK downstream and finally results in tumor proliferation and cell survival.

In conclusion, we demonstrate a novel method of using MREs in the identification of functionally relevant mRNA-microRNA interactions that can be potential targets in TNBC. Further, experimental validations of these interactions are warranted in developing novel therapeutic targets.

### Limitations of the Study

One of the limitations of this study is that although ReMIx enables identification of candidate mRNA and microRNA players via MRE analysis using RNA-Seq data, this does not establish the fact that the identified microRNAs are indeed present and expressed in the particular disease, in this case, TN tumors. ReMIx results only confirm that the sites on 3′UTR of mRNAs show distinct expression profiles in tumor and thus have the potential to be regulated by microRNAs in a disease-specific manner. To complement these results, microRNA expression profiles can be used to validate the existence of microRNAs and to check for expression correlation with the corresponding mRNA target(s) identified by ReMIx. This study is limited to microRNA-mediated interactions; however, several other mechanisms modulate gene expression. At the post-transcriptional level, the interplay of other noncoding RNAs, such as long non-coding RNAs, circular RNAs, and pseudogenes can collectively form the ceRNA network and compete with protein-coding genes for microRNA binding, thereby influencing their ultimate impact on gene expression. Also, during transcription, structural and chemical changes such as histone acetylation to determine the accessibility of chromatin domains, and DNA methylation to silence genes, are well-established modes of regulation, especially in cancer.

### Resource Availability

#### Lead Contact

Further information and requests for resources, code, and scripts should be directed to and will be fulfilled by the Lead Contact, Subbaya Subramanian (subree@umn.edu) or Asha Nair (Nair.Asha@mayo.edu).

#### Materials Availability

This study did not generate new unique reagents.

The 26 samples (13 tumor and normal-adjacent pairs) of the TCGA TNBC cohort for which REMIx results were obtained in this study are: TCGA-BH-A0B3-01A-11R-A056-07,TCGA-BH-A0B3-11B-21R-A089-07,TCGA-BH-A0BW-01A-11R-A115-07,TCGA-BH-A0BW-11A-12R-A115-07,TCGA-BH-A0E0-01A-11R-A056-07,TCGA-BH-A0E0-11A-13R-A089-07,TCGA-BH-A18Q-01A-12R-A12D-07,TCGA-BH-A18Q-11A-34R-A12D-07,TCGA-BH-A18V-01A-11R-A12D-07,TCGA-BH-A18V-11A-52R-A12D-07,TCGA-BH-A1EW-01A-11R-A137-07,TCGA-BH-A1EW-11B-33R-A137-07,TCGA-BH-A1F6-01A-11R-A13Q-07,TCGA-BH-A1F6-11B-94R-A13Q-07,TCGA-BH-A1FC-01A-11R-A13Q-07,TCGA-BH-A1FC-11A-32R-A13Q-07,TCGA-E2-A158-01A-11R-A12D-07,TCGA-E2-A158-11A-22R-A12D-07,TCGA-E2-A1L7-01A-11R-A144-07,TCGA-E2-A1L7-11A-33R-A144-07,TCGA-E2-A1LH-01A-11R-A14D-07,TCGA-E2-A1LH-11A-22R-A14D-07,TCGA-E2-A1LS-01A-12R-A157-07,TCGA-E2-A1LS-11A-32R-A157-07,TCGA-GI-A2C9-01A-11R-A21T-07,TCGA-GI-A2C9-11A-22R-A21T-07.

#### Data and Code Availability

The code for the ReMIx workflow is available through GitHub at https://github.com/nairasha/ReMIx.

## Methods

All methods can be found in the accompanying Transparent Methods supplemental file.

## Supplemental Information

Document S1. Transparent Methods, Table S1, and Figures S1–S3

Data S1. CQN (Conditional Quantile Normalization) Values for 111,521 MREs for TCGA TN (Triple-Negative) Tumor and Matched Normal-Adjacent Samples, Related to Figure 1

Data S2. CQN (Conditional Quantile Normalization) Values for 111,521 MREs for TCGA ER+ (Estrogen Receptor+) Tumor and Matched Normal-Adjacent Samples, Related to Figure 1

Data S3. CQN (Conditional Quantile Normalization) Values for 111,521 MREs for TCGA ErbB2 Overexpressed–HER2 Positive (HER2+) Tumor and Matched Normal-Adjacent Samples, Related to Figure 1

Data S4. 614 MREs with Distinct Expression in TCGA Triple-Negative Tumors Identified Using Dunnett-Tukey-Kramer Pairwise Multiple Comparison, Related to Figures 2 and S1

Data S5. 3,053 Significant and Differentially Expressed MREs Distinct to TCGA Triple-Negative Tumors Using edgeR Bioinformatics Package, Related to Figures 2 and S1

Data S6. 221 MREs that are Significant and Differential Expressed Only in TCGA Triple-Negative Tumors (Common Candidates from DTK and edgeR), Related to Figures 2 and S1

Data S7. 88 mRNAs Identified by ReMIx that Are Associated with the 221 MREs in TCGA Triple-Negative Tumors, Related to Figure 3

Data S8. 125 miRNAs Identified by ReMIx that Are Associated with the 221 MREs in TCGA Triple-Negative Tumors, Related to Figure 3

Data S9. 68 mRNAs that Are Both Differentially Expressed and Have Interacting MRE Sites for miRNA Regulation, Related to Figures 3 and S2

Data S10. 64 miRNAs that are Both Differentially Expressed and Participate in MRE Mediated Gene Expression Regulation according to ReMIx Analysis, Related to Figures 3 and S3

Data S11. Direction and Magnitude of Fold Change for 221 MREs and Their Associated Genes and microRNAs, Related to Figures 3 and S3

Data S12. GSEA (Gene Set Enrichment Analysis) Ranked by 88 mRNAs Identified to ReMIx to Have Interacting MRE Sites for miRNA Regulation, Related to Figure 4

Data S13. SPIA (Signaling Pathway Impact Analysis) on 88 mRNAs Identified by ReMIx to Have Interacting MRE Sites for miRNA Regulation, Related to Figure 4
