# Supplementary material for: Frequency of MicroRNA Response Elements Identifies Pathologically Relevant Signaling Pathways in Triple-Negative Breast Cancer
Source: iScience. 2020 Jun 6;23(6):101249. doi: 10.1016/j.isci.2020.101249 (PMC7322352; doi:10.1016/j.isci.2020.101249)
Supplement: Document S1. Transparent Methods, Table S1, and Figures S1–S3 [file mmc1.pdf]

iScience, Volume *23*

Supplemental Information

Frequency of MicroRNA Response Elements Identifies Pathologically Relevant Signaling Pathways in Triple-Negative Breast Cancer

Asha A. Nair, Xiaojia Tang, Kevin J. Thompson, Peter T. Vedell, Krishna R. Kalari, and Subbaya Subramanian

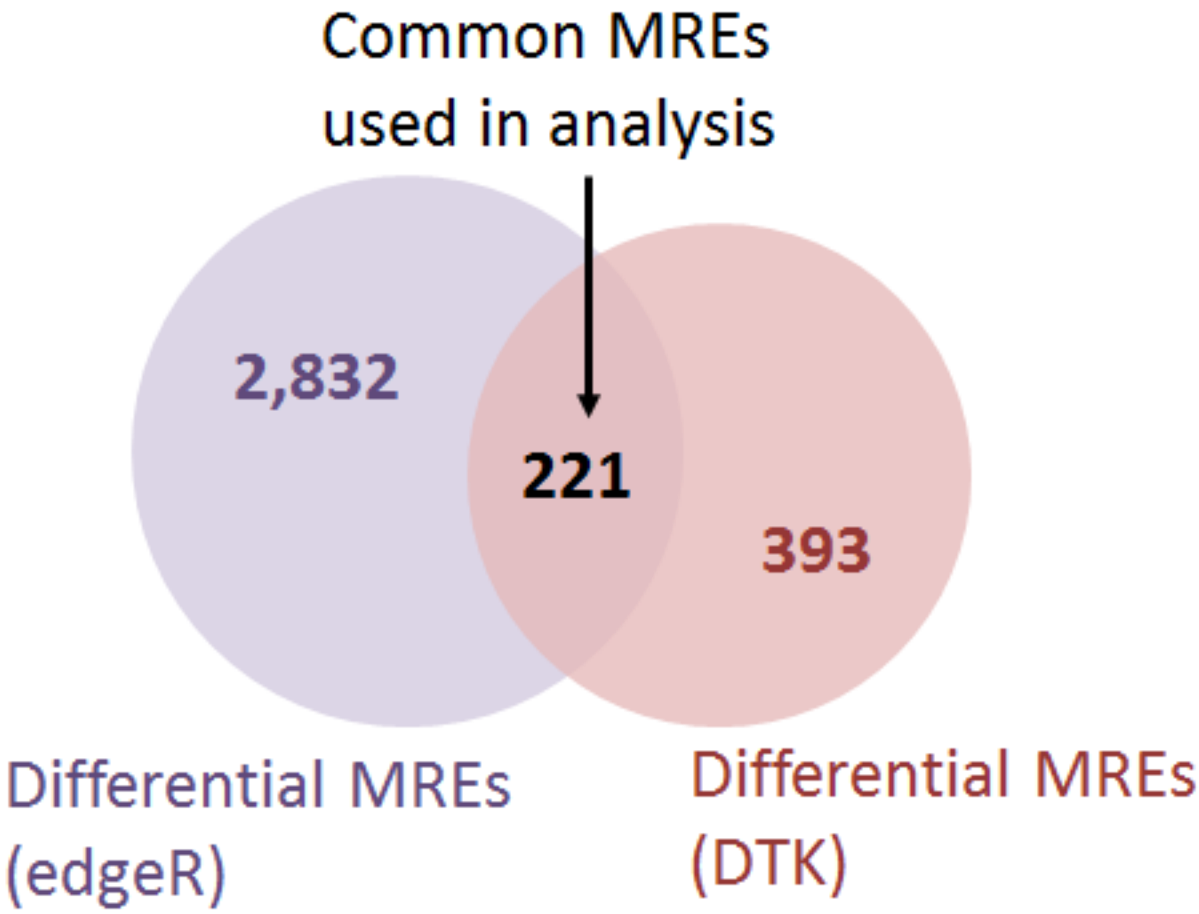

Figure S1: Common set of 221 TN tumor-specific MRE sites. Related to Figures 2 and 3

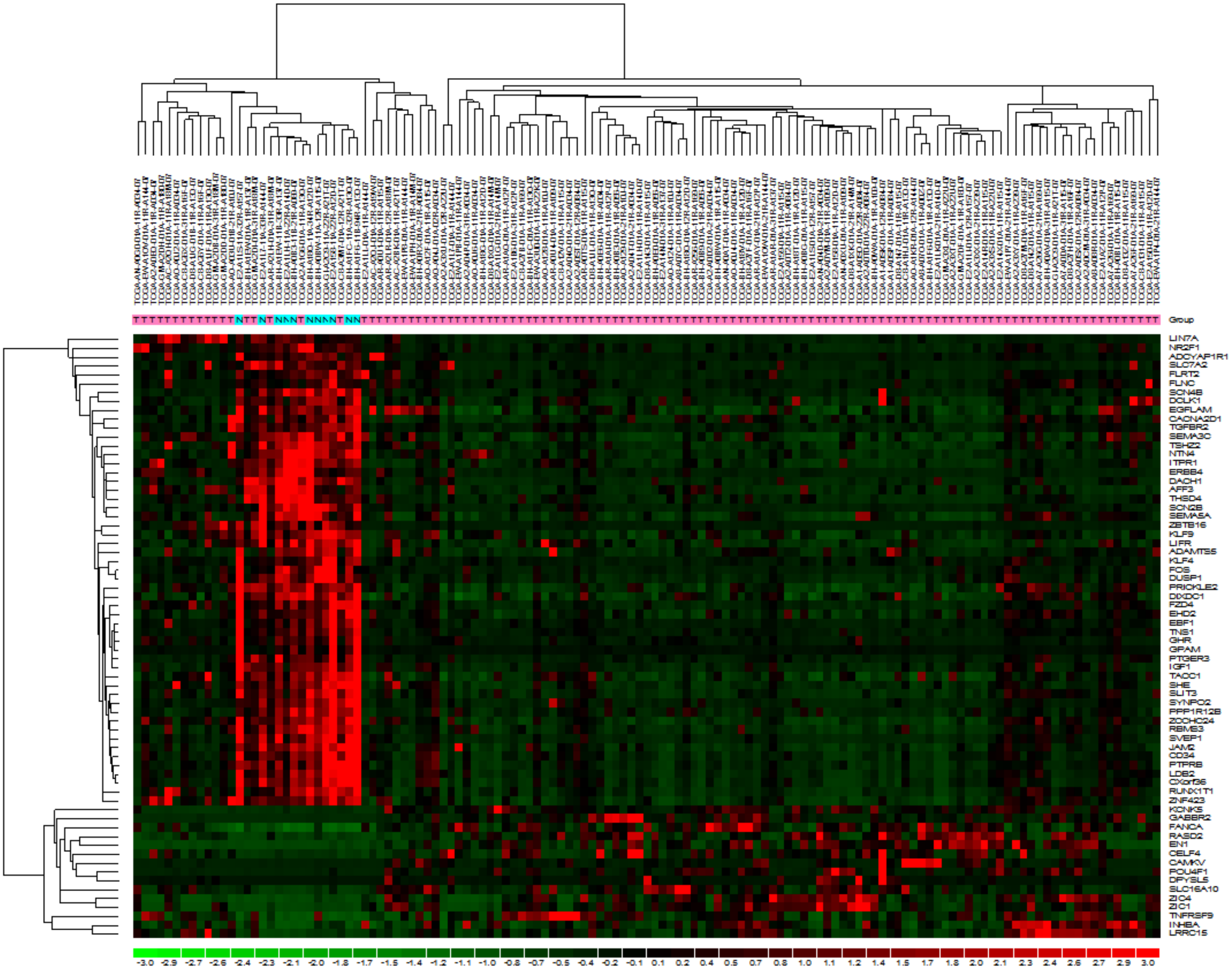

Figure S2: Differential expression of 68 mRNAs in a larger cohort of 120 TCGA-TNBC and 13 normal-adjacent samples. Related to Figure 3.

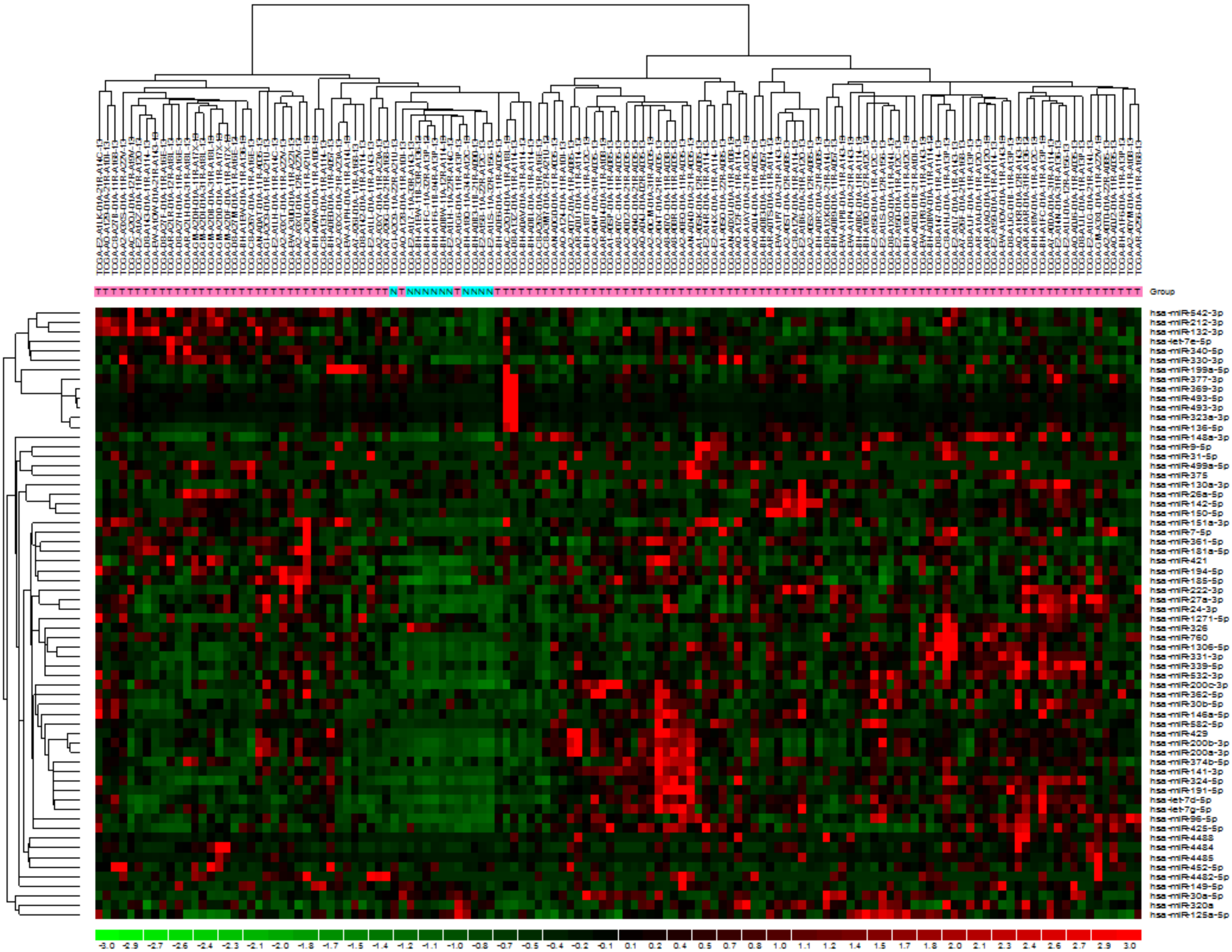

Figure S3: Heatmap listing 64 microRNAs that are both differentially expressed and participate in MRE mediated gene expression regulation. Related to Figure 3.

Table S1. TAM 2.0 results for microRNA set pathway analysis. Related to Figure 4.

Category: Disease	Count	Percent	Fold	P-value	Bonferroni	FDR
Carcinoma, Hepatocellular	60	17.7%	3.06	5.48e-28	5.73e-26	6.64e-23
Carcinoma, Gastric	55	20%	3.46	1.31e-24	1.37e-21	7.90e-22
Carcinoma, Colon	57	18%	3.13	1.48e-23	1.54e-20	5.96e-21
Carcinoma, Lung, Non-Small-Cell	47	21%	3.71	7.07e-21	7.40e-18	2.14e-18
Carcinoma, Breast	55	15.8%	2.73	3.38e-20	3.53e-17	8.18e-18
Carcinoma, Prostate	49	19%	3.30	1.34e-19	1.40e-16	2.70e-17
Neoplasms (unspecific)	44	21%	3.66	8.17e-19	8.55e-16	1.24e-17
Carcinoma, Hepatocellular (UP)	38	25%	4.44	7.82e-19	8.18e-16	1.35e-16
Glioblastoma	39	23%	3.99	1.38e-17	1.44e-14	1.86e-15
Leukemia, Myeloid, Acute	32	27%	4.65	4.34e-16	4.54e-13	4.87e-14
Glioma	41	20%	3.4	6.17e-16	6.45e-13	6.22e-14
Melanoma	33	25%	4.32	1.38e-15	1.44e-12	1.29e-13
Breast Neoplasms	34	23%	3.97	7.08e-15	7.40e-12	6.12e-13
Carcinoma, Breast, Triple Negative	14	23%	4.04	2.70e-06	2.83e-3	2.87e-5

TRANSPARENT METHODS

ReMIx – a novel methodology to compute MRE frequency from RNA-Seq data

We developed an innovative bioinformatics approach called ReMIx, which was used to quantify MRE sites at the 3'UTR regions of mRNAs from RNA-Seq data. ReMIx uses reads aligned to the 3'UTR of genes and scans them for evidence of any given MRE sequence. MRE sequences, which are complementary to the seed sequences of microRNAs, are searched in the 3'UTR of genes that are known to be associated with microRNAs (TargetScan – human version 7.0 (Agarwal et al., 2015)). A hypothetical example of this approach is illustrated in **Figure 6**. The reads aligned to 3'UTR regions of genes Gene A and Gene B are shown in Tumor and Normal-Adjacent samples. Gene A contains two MRE sites, and Gene B has one MRE site, with a common site (MRE1) in both genes. The number of reads mapped to these MRE sites are quantified for each gene and tabulated separately for Tumor and Normal-Adjacent samples. Later, MRE counts per gene are normalized and statically evaluated to identify differentially expressed MREs for downstream analysis.

MRE frequency analysis from RNA-Seq data

Seed sequences for all the conserved microRNA families (n=329) were downloaded from TargetScanHuman 7.1, and the corresponding complementary MRE sequences were derived using in-house bioinformatics scripts. **Figure 1** is a flowchart representation of the ReMIx methodology. In ReMIx, the RNA-Seq BAM files for both Tumor and Normal-Adjacent were subset to the 3'UTR regions for all genes using the SAMTools suite (Li et al., 2009). The newly obtained BAM files were converted into a FASTQ format for every gene, using the bam2fastx module from Tophat (Trapnell et al., 2009). Next, using MRE sequences of individual microRNAs and the FASTQ files for corresponding genes, the frequency of each MRE was quantified using FIMO (Grant et al., 2011). MRE sites with p-value < 0.05 were selected from the FIMO output for downstream processing. The raw MRE counts were then normalized to account for sample library size, 3'UTR length and 3'UTR GC content per gene. Finally, for every gene and for every conserved microRNA that targets the gene, the normalized MRE counts were reported in a tab-delimited format for each gene-microRNA pair in the Tumor and Normal-Adjacent cases separately.

3’UTR definitions obtained from TargetScan

Bartel’s group developed an improved quantitative model to predict canonical targeting of microRNAs to 3’UTR regions of mRNA (Agarwal et al., 2015). A combination of 14 features in the model coupled with experimental approaches such as poly(A)-position profiling by sequencing called 3P-seq was used to define 3’UTR positions of genes in the transcriptome accurately. This data, available at the TargetScan Human 7.1 database, is what was used for 3’UTR definitions of genes in the MRE analysis study.

RNA-Seq and microRNA-Seq data from TCGA

The RNA-Seq and the microRNA Sequencing fastq files for the TCGA breast cancer samples were downloaded from the TCGA Research Network (http://cancergenome.nih.gov/) using the National Cancer Institute (NCI) Genomic Data Commons (GDC) resource (https://gdc.cancer.gov/). The RNA-Seq fastq files and aligned to the hg19/NCBI 37.1 human reference genome using the MAP-RSeq workflow (Kalari et al., 2014) and the microRNA fastq files were aligned using the CAP-miRSeq workflow (Sun et al., 2014). The normalized microRNA counts from CAP-miRSeq were used to obtain the microRNA expression values in the TNBC samples.

The differential expression analysis of the RNA-Seq data for the TNBC tumor and normal-adjacent pairs were obtained using the bioinformatics R package edgeR (Robinson et al., 2010). The statistical significance threshold used was FDR < 5% and log2FC |2|. For these 13 pairs of TNBC cases, differential expression analysis of the microRNA sequencing data was performed using the R bioinformatics package called limma (Ritchie et al., 2015). The statistical threshold used to identify significantly differential expressed microRNAs was adjusted p-value <0.05.

MRE site evaluation and activated pathway identification

Evaluation of MRE sites that represented distinct and TNBC-specific expression as opposed to ER+ and HER2+ subtypes and normal-adjacent cases were obtained using the R package Dunnett-Tukey-Kramer Pairwise Multiple Comparison Test Adjusted for Unequal Variances and Unequal Sample Sizes. Statistically significant MRE sites were selected using p-value cut-off < 0.05. The bioinformatics R

package edgeR (Robinson et al., 2010) was used to obtained differentially expressed MREs between TN tumors and matched normal-adjacent pairs at FDR <5% and log2FC |2|.

Pathway analysis for canonical pathways

The microRNA set analysis tool called TAM2.0 was used to identify cancer-related pathways for the 125 microRNAs. Likewise, enriched canonical pathway analysis for 88 genes was performed using KEGG and Reactome functional databases. Open source analysis toolkit WebGestalt (Wang et al., 2017) was also used for pathway identification by using the option to perform Gene set enrichment analysis (GSEA). Identification of the relevance and activation/inhibition status of pathways was evaluated using the R package called Signaling Pathway Impact Analysis (SPIA). The Bioconductor R package called Pathview (Luo et al., 2017) was used to map the gene expression data from TNBC and visualize the MAPK pathway using the KEGG-based network model of this pathway.

Statistical Methods

The various statistical tests performed in this study are summarized as follows –

1. MRE selection and normalization: (a) raw counts for MRE sites were quantified using the Find Individual Motif Occurrences (FIMO) tool and MREs with p-value < 0.05 were selected for downstream analysis, (b) raw counts were normalized using conditional quantile normalization (CQN) to account for sample library size, 3'UTR length and 3'UTR GC content per gene.
2. Identification of TNBC specific MREs: (a) first, Dunnett-Tukey-Kramer (DTK) pairwise multiple comparison statistical test was applied to tumor and normal-adjacent of all subtypes – TNBC, ER+ and Her2+ (6 groups in total) to obtain 614 TNBC MREs at p-value < 0.05, (b) second, edgeR bioinformatics package was applied to TNBC tumor and normal-adjacent samples to identify 3,053 MREs (FDR < 5% and log2FC |2|), (c) finally, union of the DTK and edgeR results were used to arrive at 221 MREs.
3. RNASeq data of TNBC tumor and normal-adjacent samples were compared for differential expression analysis using the edgeR package to obtain 2,250 genes at the statistical significance threshold of FDR (false discovery rate) < 5% and log2 fold change > 2 or < -2.

4. The microRNA sequencing data of TNBC tumor and normal-adjacent samples were compared for differential expression analysis using the limma package to obtain 778 microRNAs at the statistical significance threshold of adjusted p-value < 0.05.
5. The microRNA pathway analysis was performed using the TAM 2.0 tool for enrichment analysis of the 125 microRNAs found by ReMIx. Out of 125 microRNAs, 14 microRNAs were associated with the upregulation of disease at FDR < 2.87e-5, 55 microRNAs reported in breast carcinoma studies at FDR < 8.18e-18, and 34 microRNAs in breast neoplasms at (FDR < 6.12e-13).
6. The mRNA pathway analysis was performed using (a) gene set enrichment analysis (GSEA) – with the FDR values reported in Supplemental File 12, (b) SPIA - pGFdr and pGFWER are the False Discovery Rate and Bonferroni adjusted global p-values reported in Supplemental file 13.

Supplemental References

Agarwal, V., Bell, G.W., Nam, J.W., and Bartel, D.P. (2015). Predicting effective microRNA target sites in mammalian mRNAs. Elife *4*.

Grant, C.E., Bailey, T.L., and Noble, W.S. (2011). FIMO: scanning for occurrences of a given motif. Bioinformatics *27*, 1017-1018.

Kalari, K.R., Nair, A.A., Bhavsar, J.D., O'Brien, D.R., Davila, J.I., Bockol, M.A., Nie, J., Tang, X., Baheti, S., Doughty, J.B., *et al.* (2014). MAP-RSeq: Mayo Analysis Pipeline for RNA sequencing. BMC Bioinformatics *15*, 224.

Li, H., Handsaker, B., Wysoker, A., Fennell, T., Ruan, J., Homer, N., Marth, G., Abecasis, G., Durbin, R., and Genome Project Data Processing, S. (2009). The Sequence Alignment/Map format and SAMtools. Bioinformatics *25*, 2078-2079.

Luo, W., Pant, G., Bhavnasi, Y.K., Blanchard, S.G., Jr., and Brouwer, C. (2017). Pathview Web: user friendly pathway visualization and data integration. Nucleic Acids Res *45*, W501-W508.

Ritchie, M.E., Phipson, B., Wu, D., Hu, Y., Law, C.W., Shi, W., and Smyth, G.K. (2015). limma powers differential expression analyses for RNA-sequencing and microarray studies. Nucleic Acids Res *43*, e47.

Robinson, M.D., McCarthy, D.J., and Smyth, G.K. (2010). edgeR: a Bioconductor package for differential expression analysis of digital gene expression data. Bioinformatics *26*, 139-140.

Sun, Z., Evans, J., Bhagwate, A., Middha, S., Bockol, M., Yan, H., and Kocher, J.P. (2014). CAP-miRSeq: a comprehensive analysis pipeline for microRNA sequencing data. BMC Genomics *15*, 423.

Trapnell, C., Pachter, L., and Salzberg, S.L. (2009). TopHat: discovering splice junctions with RNA-Seq. Bioinformatics *25*, 1105-1111.

Wang, J., Vasaikar, S., Shi, Z., Greer, M., and Zhang, B. (2017). WebGestalt 2017: a more comprehensive, powerful, flexible and interactive gene set enrichment analysis toolkit. Nucleic Acids Res *45*, W130-W137.
